# Associations Between Life Satisfaction and Sleep Quality Among Older Adults in China: Mediating Roles of Psychological Resilience and Anxiety and a Moderating Role of Chronic Disease

**DOI:** 10.3390/healthcare14060787

**Published:** 2026-03-20

**Authors:** Ziting Zhao, Shiyi Xu, Zhijie Cai, Yinliang Ge, Ziwei Zhang, Xuebin Qiao, Kan Tian

**Affiliations:** 1 School of Health Economics and Management, Nanjing University of Chinese Medicine, Nanjing 210023, China; 2 School of Elderly Care Services and Management, Nanjing University of Chinese Medicine, Nanjing 210023, China; 3 School of Medicine, Nanjing University of Chinese Medicine, Nanjing 210023, China; 4 Jiangsu Research Center for Major Health Risk Management and TCM Control Policy, Nanjing University of Chinese Medicine, Nanjing 210023, China; 5 Institute of Elderly Care Services and Management, Nanjing University of Chinese Medicine, Nanjing 210023, China

**Keywords:** life satisfaction, sleep quality, psychological resilience, anxiety, chronic disease

## Abstract

**Background**: Poor sleep quality is common in older adults and closely tied to emotional well-being. While life satisfaction has been linked to sleep outcomes, the psychological pathways remain underexplored. **Purpose**: This study examines whether psychological resilience and anxiety symptoms mediate the relationship between life satisfaction and sleep quality among older adults, and whether chronic disease moderates these pathways. **Methods**: Data were drawn from the 2018 wave of the Chinese Longitudinal Healthy Longevity Survey, including 3089 community-dwelling adults aged 65 years and older. Sleep quality and life satisfaction were measured using validated single-item indicators. Psychological resilience was assessed using five self-reported items capturing adaptive functioning, and anxiety symptoms were measured using the 7-item Generalized Anxiety Disorder Scale. A parallel mediation model and a moderated mediation model were tested using SPSS with bootstrapping procedures (5000 samples). **Results**: Life satisfaction was significantly associated with sleep quality among older adults. This association was partially mediated by both psychological resilience (indirect association = −0.0100, 95% CI [−0.0163, −0.0045]) and anxiety symptoms (indirect association = −0.0356, 95% CI [−0.0483, −0.0238]). The direct association remained significant (β = −0.2399, *p* < 0.001), indicating a partial mediation pattern. Furthermore, in the moderated mediation model, chronic disease moderated the association between anxiety symptoms and sleep quality, whereas the indices of moderated mediation were not significant. **Conclusions**: Life satisfaction was associated with sleep quality, with psychological resilience and anxiety symptoms accounting for part of this association. Although chronic disease strengthened the association between anxiety symptoms and poorer sleep quality, the overall moderated mediation effect was not significant.

## 1. Introduction

As the global population continues to age, promoting the health and well-being of older adults has become a critical public health priority [1]. Among the various challenges that accompany aging, sleep disturbances have emerged as one of the most prevalent and persistent health problems in later life [2]. Epidemiological studies suggest that nearly half of older adults worldwide experience some form of sleep difficulty, including difficulties falling asleep, frequent nocturnal awakenings, and poor overall sleep quality [3]. In China, national surveys estimate that more than 38% of older adults report poor sleep quality—a figure that has steadily increased with rising life expectancy and population aging [4].

Sleep quality is more than a physiological outcome; it is a multidimensional health indicator closely linked to emotional regulation, cognitive performance, immune functioning, and overall life satisfaction [5,6]. Poor sleep in late life has been associated with heightened risks of depression, cardiovascular disease, cognitive decline, impaired daily functioning, and premature mortality [7,8,9]. The pervasive impact of sleep problems underscores the importance of identifying modifiable psychosocial factors associated with sleep quality in older adults [10,11]. Despite the growing emphasis on pharmacological and behavioral interventions, there is increasing recognition that subjective psychological experiences—such as one’s perception of life satisfaction—may play a foundational role in shaping sleep outcomes.

From a broader theoretical standpoint, understanding how subjective well-being is associated with sleep aligns with the stress-coping framework, which emphasizes the role of psychological resources and emotional vulnerabilities in shaping health trajectories [12,13]. Within this framework, sleep is not simply an outcome of biological aging but a sensitive indicator of how older adults psychologically adapt to life changes and chronic stressors [14,15]. Therefore, elucidating the psychosocial pathways that underlie sleep quality in later life is essential for developing person-centered interventions that promote healthy aging at both individual and population levels [16].

### 1.1. The Mediating Role of Psychological Resilience

Among the psychosocial determinants of sleep, life satisfaction has received growing attention as a key component of subjective well-being in older adulthood. Conceptually defined as an individual’s global cognitive evaluation of their quality of life, life satisfaction reflects long-term psychological contentment and has been linked to a wide range of health outcomes [17]. Prior research has consistently demonstrated that higher levels of life satisfaction are associated with reduced risk of chronic illness, enhanced emotional well-being, and greater longevity [18,19]. In the context of sleep, individuals who report higher life satisfaction tend to experience better subjective sleep quality, longer sleep duration, and fewer insomnia symptoms [20].

These associations may be particularly salient in older adults, who often face accumulated stressors such as declining physical function, social loss, or financial insecurity [21]. In such contexts, life satisfaction may serve as a psychological buffer that fosters emotional regulation and mitigates the physiological stress response that disrupts sleep [22]. However, despite well-documented correlations between life satisfaction and sleep outcomes, the psychological processes that may help explain this association remain insufficiently understood. Existing studies have largely treated life satisfaction as a distal predictor of sleep quality, without addressing the intermediary psychological processes that might help explain the association [23].

To address this gap, the present study considers psychological resilience as a potential mediating variable. Resilience reflects an individual’s capacity to adapt positively in the face of adversity, enabling effective coping and emotional regulation. Among older adults, resilience plays a pivotal role in navigating age-related losses and maintaining a sense of control and purpose despite life challenges. Thus, life satisfaction may be associated not only with sleep quality directly but also with psychological resilience, which may in turn be associated with better sleep quality. By incorporating resilience as a mediating factor, this study seeks to examine whether subjective well-being is associated with adaptive emotional functioning and better sleep in later life.

### 1.2. The Mediating Role of Anxiety Symptoms

In contrast to psychological resilience as a protective mechanism, anxiety symptoms may represent a vulnerability pathway that helps explain the association between life satisfaction and sleep quality in older adults [24]. Anxiety is a prevalent psychological condition in late life, often presenting as excessive worry, nervous tension, and heightened physiological arousal [25]. Unlike depression, which is primarily associated with low mood and disengagement, anxiety involves sustained hypervigilance and anticipatory stress that can disrupt both emotional stability and somatic functioning [26]. Empirical evidence has shown that elevated anxiety symptoms are strongly correlated with poor sleep quality, including difficulties initiating and maintaining sleep, reduced sleep efficiency, and fragmented sleep architecture [27].

Older adults with low life satisfaction may be particularly vulnerable to anxiety, as dissatisfaction with one’s life circumstances may be linked to persistent rumination, loss of perceived control, and greater sensitivity to daily stressors [28]. From a stress-process perspective, diminished life satisfaction may also be associated with greater chronic psychological burden and less adaptive stress responses, which may in turn relate to higher anxiety levels. Anxiety may further be associated with poorer sleep through cognitive and physiological correlates, such as worry-related intrusive thoughts, increased heart rate, and muscle tension [29,30].

Despite these theoretical linkages, few studies have explicitly examined anxiety as a mediator in the association between life satisfaction and sleep among older adults. By including anxiety symptoms as a risk-oriented mediator, the current study aims to capture the emotional costs of low subjective well-being and to examine whether psychological distress may help explain poorer sleep. This approach complements the resilience pathway and provides a dual-process model that integrates both emotional resources and liabilities in explaining sleep outcomes in later life.

### 1.3. The Moderating Role of Chronic Disease

Chronic disease is a common and consequential condition among older adults, with far-reaching implications for both physical functioning and psychological well-being [31]. Age-related illnesses such as cardiovascular disease, diabetes, arthritis, and chronic pain syndromes are not only biologically progressive but also psychologically taxing, often accompanied by long-term treatment burdens, mobility limitations, and increased dependence on others [32]. Numerous studies have shown that chronic conditions are significantly associated with heightened anxiety, depressive symptoms, and poor sleep quality in late life [33]. These illnesses can interfere with circadian rhythms, increase physical discomfort, and amplify emotional reactivity, all of which may be related to sleep disturbances [34,35].

From a psychosocial perspective, chronic disease may be directly associated with poorer sleep quality and may also alter the strength of psychological processes associated with sleep. Specifically, physical illness may weaken the benefits of positive psychological resources such as resilience by overwhelming the individual’s coping capacity [35]. At the same time, chronic disease may be associated with stronger links between negative emotional states and poor sleep, potentially because physical symptoms and health-related uncertainty are related to higher physiological arousal and greater cognitive rumination [32]. The interplay between physical and psychological factors is therefore critical in understanding how the association between life satisfaction and sleep may differ among individuals with differing health profiles.

In this context, chronic disease is conceptualized as a potential moderator of the indirect association linking life satisfaction to sleep quality through psychological resilience and anxiety symptoms [33]. By testing moderated mediation pathways, the current study seeks to determine whether the emotional mechanisms underlying sleep quality differ across health status subgroups. This approach not only contributes to a more nuanced understanding of individual variability in sleep outcomes but also offers practical implications for tailoring interventions to older adults who are most at risk due to their chronic health burden.

### 1.4. Theoretical Integration and Research Hypotheses

Drawing on the stress-coping framework and existing empirical evidence, the present study proposes a theoretically integrated model to explain the association between life satisfaction and sleep quality among older adults through both protective and risk-related emotional processes. Within this model, psychological resilience is positioned as a positive psychological resource that enables older individuals to adapt to adversity and maintain emotional balance, while anxiety symptoms represent a negative emotional state that disrupts cognitive and physiological functioning. These dual mediating pathways reflect the coexistence of strength- and vulnerability-based pathways through which subjective well-being may be linked to sleep outcomes in late life.

In addition to these mediators, the model incorporates chronic disease as a potential moderator that may alter the strength of the indirect association. Chronic illness may strengthen the association between anxiety and sleep and weaken the resilience-related pathway, thus introducing individual variability into the effectiveness of psychological coping mechanisms. This moderated mediation approach provides a more differentiated understanding of how life satisfaction is associated with sleep quality across diverse health contexts.

Within this theoretical framework, the proposed ordering is guided by the Broaden-and-Build Theory, which suggests that positive evaluations of life help individuals build enduring psychological resources. Accordingly, higher life satisfaction is expected to be linked to greater psychological resilience and lower anxiety symptoms, both of which are associated with better sleep quality. In the present study, this ordering is used as a theory-driven framework for understanding the association between life satisfaction and sleep quality among older adults.

Based on this conceptual framework, the following hypotheses were proposed:

 **Hypothesis H1:** *Life satisfaction is positively associated with sleep quality among older adults.*

 **Hypothesis H2:** *Psychological resilience may mediate the association between life satisfaction and sleep quality.*

 **Hypothesis H3:** *Anxiety symptoms may mediate the association between life satisfaction and sleep quality.*

 **Hypothesis H4:** *The indirect association between life satisfaction and sleep quality through psychological resilience and anxiety symptoms may vary by chronic disease status.*

**Figure 1:**
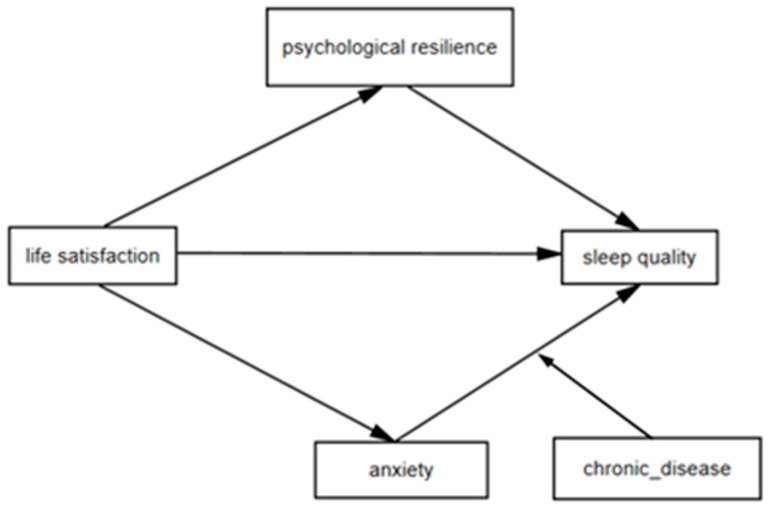
Hypothesis testing model.

Figure 1 illustrates the proposed conceptual model, integrating both parallel mediation and moderated mediation components to capture the complexity of psychosocial associations with sleep in older adulthood.

## 2. Materials and Methods

### 2.1. Participants

The data were derived from the Chinese Longitudinal Healthy Longevity Survey (CLHLS), which was a nationwide survey project conducted by the Center for Healthy Aging and Development at the National School of Development, Peking University. The present study used data from the 2018 wave of the CLHLS, which covered more than 500 sample sites in 22 of China’s 31 provinces, with over 15,000 participants aged 65 years and older.

The survey collected comprehensive information on sociodemographic characteristics, health behaviors, mental health, chronic conditions, and family living arrangements. All data were obtained through structured face-to-face interviews. Participants were asked about their gender, age, residence, marital status, education level, income sufficiency, and whether they lived with family members. Sleep quality, chronic disease status, and psychological well-being were also assessed. Mental health was measured using the 10-item Center for Epidemiological Studies Depression Scale (CES-D-10) and the 7-item Generalized Anxiety Disorder Scale (GAD-7).

This study aimed to examine the association between life satisfaction and sleep quality among older adults, with psychological resilience and anxiety symptoms as mediators, and chronic disease as a moderator. Inclusion criteria were: (1) aged 65 years or older; (2) living in the community (not in nursing institutions); and (3) having complete responses for key study variables. Participants with missing values greater than 5% or with extreme or invalid responses were excluded. For variables with less than 5% missing data, mean imputation was applied. After data cleaning, a total of 3089 older adults were included in the final analytic sample. The participant selection process is illustrated in Figure 1.

### 2.2. Measures

#### 2.2.1. Dependent Variables

Sleep quality was assessed using the question “How is your sleep quality now?” Responses ranged from 1 (very good) to 5 (very poor). For consistency with other variables, scores were reverse-coded such that higher values indicated better subjective sleep quality [36]. This single-item self-rated sleep quality measure has been widely used in CLHLS-based studies to characterize older adults’ subjective sleep status [4,37,38]. Note that the original five-point sleep-quality item was reverse-scored so that higher scores indicate better subjective sleep quality; thus, a negative β in the regression tables represents the adverse association of poorer sleep. Notably, sleep quality in this study was assessed as a subjective self-rated indicator and thus may not fully correspond to objective or physiological sleep parameters.

#### 2.2.2. Independent Variables

Life satisfaction was measured using the item “How do you rate your life at present?” from the CLHLS 2018 wave. Responses were originally rated on a five-point Likert scale (1 = very good, 2 = good, 3 = neutral, 4 = bad, 5 = very bad) and were reverse-coded in the present study so that higher scores reflected greater life satisfaction (1 = very bad to 5 = very good). This single-item measure of life satisfaction has been widely used in prior studies among older Chinese adults [39,40,41], and methodological research has also supported the validity of single-item life satisfaction measures in large-scale survey settings [42].

#### 2.2.3. Mediating Variables

Psychological resilience was measured by five items derived from the CLHLS 2018, which capture cognitive, emotional, and behavioral adaptability. Respondents were asked: (1) Do you always look on the bright side of things? (2) Do you feel the older you get, the more useless you are and do you have trouble doing anything? (3) Do you often feel lonely and isolated? (4) Do you often feel fearful or anxious? (5) Can you make your own decisions concerning your personal affairs? Responses ranged from 1 (always) to 5 (never). Items (1) and (5) were reverse-coded to ensure consistency, and all five items were summed to generate a composite resilience score, ranging from 5 to 25, with higher scores indicating greater psychological resilience. Although the CLHLS was not originally designed to measure psychological resilience, previous studies have provided support for the conceptual validity of these items. The internal consistency of the scale in this study was high (Cronbach’s alpha = 0.891). Exploratory factor analysis of these five items supported a one-factor structure, with an overall Kaiser-Meyer-Olkin (KMO) value of 0.6107.

Anxiety symptoms were measured using the Generalized Anxiety Disorder Scale (GAD-7), which consists of seven items assessing the frequency of anxiety-related experiences over the past two weeks. Each item was rated on a four-point scale: 0 = never, 1 = several days, 2 = more than half the days, 3 = nearly every day. Total scores ranged from 0 to 21, with higher scores reflecting more severe anxiety. A cut-off score of 5 was used to indicate mild anxiety. This threshold represents at least mild anxiety symptoms and is consistent with the standard severity classification of the GAD-7 [43]. In addition, prior CLHLS-based studies have also adopted this threshold when defining the presence of anxiety symptoms [44,45]. The GAD-7 has been validated among older Chinese adults and demonstrated good internal consistency in this study (Cronbach’s alpha = 0.919).

#### 2.2.4. Moderator and Covariates

Based on previous studies, the control variables were divided into the following three categories: sociodemographic characteristics, socioeconomic status (SES), and living arrangements. The first group of control variables included gender (female = 1), age, and current marital status (married = 1). Residence was classified as rural or urban, with urban coded as 1. Age was grouped into three life-stage categories: 65–74 years (coded as 1), 75–84 years (coded as 2), and 85 years and above (coded as 3).

The second group of covariates reflected socioeconomic status. Education level was dichotomized as literate (1) versus illiterate (0). Income sufficiency was assessed by asking whether respondents considered their financial resources sufficient to meet daily needs (sufficient = 1).

The third group included a living arrangement variable, indicating whether the respondent was currently living with family members (yes = 1).

Depression and cognitive function were not included as covariates in the primary models, as the present study focused on resilience and anxiety as the theory-driven psychological mechanisms.

These control variables were included in all regression models to account for potential confounding factors that might be associated with sleep quality among older adults.

The respondents were asked whether they had any physician-diagnosed chronic diseases at the time of the survey. In this study, chronic disease was operationalized as a binary variable based on respondents’ self-report of having at least one physician-diagnosed chronic condition, including hypertension, diabetes, heart disease, stroke or cerebrovascular disease, chronic bronchitis or emphysema or asthma, tuberculosis, cataract, glaucoma, cancer or malignant tumor, Parkinson’s disease, arthritis or rheumatism, gastric ulcer, and chronic kidney disease. Participants who reported having any chronic illness were coded as 1, and those without chronic illness were coded as 0. This variable was used as a moderator in the analysis.

### 2.3. Statistical Analyses

All statistical analyses were conducted using IBM SPSS Statistics version 26.0. Prior to hypothesis testing, descriptive statistics and Pearson correlation analyses were performed to examine the distribution and bivariate relationships of all key variables. To reduce the potential for multicollinearity and facilitate the interpretation of interaction terms, all continuous variables were standardized before analysis. Categorical variables, including chronic disease status, were dummy coded. Normality was evaluated using skewness and kurtosis statistics together with visual inspection of histograms and Q–Q plots, and no substantial deviation from normality was observed.

To assess potential common method bias, Harman’s single-factor test was conducted using principal component analysis. Five factors with eigenvalues greater than 1 were extracted, and the first unrotated factor accounted for 30.76% of the total variance, which is below the commonly used threshold of 40%, suggesting that common method bias was not a major concern in this study.

Subsequently, a series of hierarchical regression models were estimated to test the direct association between sleep quality and life satisfaction, followed by the inclusion of mediators and interaction terms to examine the conditional association. Control variables included gender, age group, marital status, urban residence, literacy, perceived income sufficiency, and co-residence with family members.

To formally test the hypothesized mediation relationships, the PROCESS macro (Version 4.1) developed by Hayes was employed. A parallel mediation model (Model 4) was first used to evaluate whether psychological resilience and anxiety symptoms independently mediated the relationship between life satisfaction (independent variable, X) and sleep quality (dependent variable, Y). The significance of the indirect association was assessed using 5000 bias-corrected bootstrap samples and 95% confidence intervals (CIs).

To further explore the conditional nature of the mediation paths, a moderated parallel mediation model was tested, in which chronic disease (W) was specified as a moderator of both the direct and indirect associations of life satisfaction on sleep quality via the two mediators. The significance of moderated mediation relationships was evaluated by examining the index of moderated mediation and corresponding bootstrap CIs. When significant interaction association were detected, the Johnson–Neyman technique and simple slope analyses were applied to visualize the region of significance and the directionality of moderation.

A two-tailed *p*-value of <0.05 was considered statistically significant for all analyses.

## 3. Results

### 3.1. Common Method Bias and Test

Because the data in this study were obtained through self-reported questionnaires, the possibility of common method bias (CMB) was considered. To assess this, Harman’s single-factor test was conducted using principal component analysis (PCA) on all key study variables. The analysis extracted five factors with eigenvalues greater than 1. The first unrotated factor accounted for 30.76% of the total variance, which is below the commonly accepted threshold of 40%. Therefore, common method bias was not considered a serious concern in this study.

### 3.2. Descriptive Statistics

A total of 3089 older adults were included in this study. Among them, females accounted for 56.0% of the sample. The majority were married (56.0%) and reported living with family members (76.0%), while 38.0% resided in urban areas. Regarding socioeconomic characteristics, 87.0% of participants reported sufficient income to meet daily needs, and 58.0% were literate.

In terms of health-related variables, a high proportion (86.0%) of the participants reported having chronic diseases. The mean standardized anxiety score (GAD-7) was −0.04 (SD = 0.94), suggesting an overall low level of anxiety symptoms in the sample, although individual differences were evident.

For the core variables in the study, the mean score for sleep quality (Y) was 3.52 (SD = 0.99), indicating a moderate level of perceived sleep quality. The average score for psychological resilience (M) was −0.37 (SD = 0.69), standardized based on sample distribution. The life satisfaction score (X) had a mean of 2.09 (SD = 0.81), suggesting that the overall life satisfaction level among these older adults was moderate to low. The results are presented in Table 1.

### 3.3. The Parallel Mediation Relationship Model

To examine whether psychological resilience and anxiety symptoms mediate the relationship between life satisfaction and sleep quality among older adults, a parallel mediation model was constructed using PROCESS Model 4 with 5000 bootstrap samples. After controlling for gender, age group, marital status, cohabitation status, urban residence, literacy, and household income, the model yielded several key findings.

The total association of life satisfaction with sleep quality was statistically significant (β = −0.2855, SE = 0.0218, *p* < 0.001). After including the mediators, the direct association remained significant (β = −0.2399, SE = 0.0215, *p* < 0.001), indicating partial mediation.

As shown in Table 2, both psychological resilience and anxiety symptoms significantly mediated this relationship. Specifically, the indirect association through psychological resilience was −0.0100 (Boot SE = 0.0030, 95% CI [−0.0163, −0.0045]), while the indirect association through anxiety symptoms was −0.0356 (Boot SE = 0.0063, 95% CI [−0.0483, −0.0238]). The total indirect association was −0.0456 (Boot SE = 0.0070, 95% CI [−0.0598, −0.0320]). The contrast between the two mediators was also significant (contrast = 0.0256, 95% CI [0.0122, 0.0395]), suggesting that anxiety symptoms showed a stronger mediating pathway than psychological resilience.

These findings suggest that life satisfaction was significantly associated with sleep quality among older adults, and that this association may be explained by psychological resilience and anxiety symptoms, with the latter showing a stronger indirect association.

### 3.4. Tests for Moderating Relationship of Chronic Disease

To examine whether chronic disease moderated the indirect association of life satisfaction on sleep quality via psychological resilience and anxiety symptoms, we employed Model 14 of the PROCESS. All variables were standardized before analysis. The results are presented in Table 3.

Life satisfaction was significantly associated with sleep quality (β = −0.2413, *p* < 0.001), psychological resilience (β = 0.1007, *p* < 0.001), and anxiety symptoms (β = 0.1535, *p* < 0.001). The interaction between anxiety symptoms and chronic disease was significantly associated with sleep quality (β = −0.1214, *p* = 0.018), indicating that chronic disease moderated the relationship between anxiety and sleep quality. However, the interaction between psychological resilience and chronic disease was not significant (β = 0.0466, *p* = 0.522).

**Figure 2:**
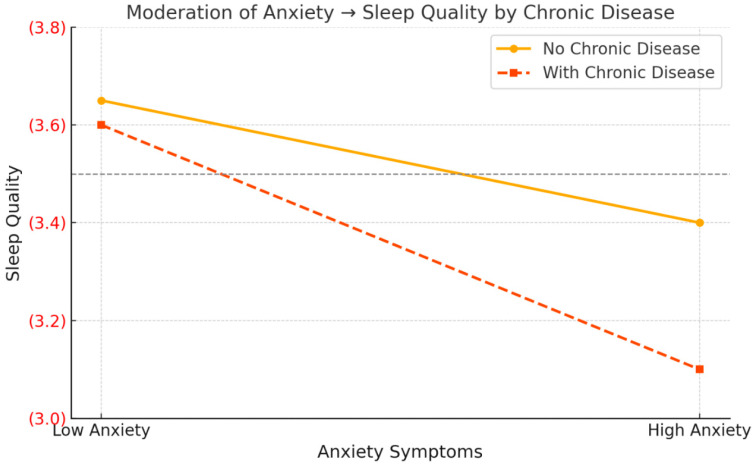
Moderating relationship of chronic disease on the relationship between anxiety symptoms and sleep quality. For individuals with chronic disease, higher anxiety was associated with a sharper decline in sleep quality.

Simple slope analysis (Figure 2) revealed that the negative association of anxiety on sleep quality was stronger with high chronic disease (β = −0.2478, t = −12.71, *p* < 0.001) and weaker with low chronic disease (β = −0.1265, t = −2.63, *p* = 0.009).

The index of moderated mediation was non-significant for both pathways: anxiety path (−0.0186, 95% CI [−0.0367, 0.0004]) and resilience path (0.0047, 95% CI [−0.0099, 0.0200]). These results indicate that the association between anxiety symptoms and sleep quality was stronger among older adults with chronic disease; however, the indices of moderated mediation were not significant, indicating no evidence for significant moderated mediation of the indirect associations.

## 4. Discussion

This study aimed to explore the psychological pathways that may underlie the association between life satisfaction and sleep quality among older adults, focusing on the mediating roles of psychological resilience and anxiety symptoms, as well as the moderating role of chronic disease. The findings supported the direct and mediating associations, whereas evidence for moderated mediation was limited.

First, life satisfaction was significantly associated with sleep quality among older adults. Second, both psychological resilience and anxiety symptoms served as significant mediators of the association between life satisfaction and sleep quality. Importantly, anxiety symptoms exerted a stronger mediating relationship than psychological resilience, highlighting the salience of emotional distress in shaping sleep experiences. Finally, chronic disease was found to moderate the anxiety pathway: the negative association between anxiety and sleep was significantly stronger among those with chronic illness, whereas no such moderating relationship was observed in the resilience pathway.

Together, these results suggest two psychological pathways that may help explain the association between life satisfaction and sleep quality—via psychological resilience and anxiety symptoms. Chronic disease appeared to moderate the anxiety–sleep association, but evidence for overall moderated mediation was not supported. This integrated model provides a nuanced understanding of the psychosocial processes influencing sleep in aging populations.

The current findings align with the broader literature in positive psychology, which posits that life satisfaction—an integral component of subjective well-being—has been consistently associated with a range of favorable health outcomes, including sleep quality [46,47]. From the perspective of affective regulation, individuals with higher life satisfaction are less prone to rumination and negative mood states before bedtime, both of which are well-documented disruptors of sleep [30]. Furthermore, cognitive appraisal theory suggests that satisfied individuals are more likely to engage in constructive meaning-making, perceive their environment as controllable, and maintain emotional stability, which collectively facilitate more restorative sleep [48,49].

In this study, life satisfaction had a significant total association with sleep quality, and this association remained robust even after accounting for mediating variables. These findings underscore the importance of fostering subjective well-being as part of interventions aimed at improving sleep health in later life. Moreover, by using a large, representative sample of Chinese older adults, this study provides culturally specific evidence supporting the universality of the life satisfaction–sleep quality link, while also expanding the positive psychology framework to aging contexts within non-Western societies.

The findings of this study suggest that psychological resilience operates as a key protective factor in the relationship between life satisfaction and sleep quality. When older adults feel more satisfied with their lives, they are more likely to appraise stressors as manageable and maintain a sense of control and emotional stability. These adaptive appraisals may be associated with greater resilience, which, in turn, helps downregulate physiological arousal and limit the cognitive intrusions that typically disrupt sleep. Within this framework, resilience functions as an internal resource that converts positive cognitive evaluations of life into greater sleep efficiency and continuity.

Importantly, this pattern remained after accounting for structural disadvantages such as low income, illiteracy, or living without family, which is consistent with the possibility that resilience may attenuate the association among subjective dissatisfaction, objective stress exposure, and sleep quality [50]. These findings reinforce the theoretical proposition that resilience is not merely the absence of vulnerability, but a dynamic capacity to preserve psychological balance under cumulative stress [51]. The ability to mobilize psychological resilience in later life may be particularly crucial given age-related losses in health, autonomy, and social roles. As such, enhancing resilience should be considered a viable strategy for promoting restorative sleep in geriatric care, especially when life satisfaction cannot be rapidly improved through structural interventions [10,52].

While psychological resilience represents a compensatory mechanism, anxiety symptoms embody the risk-laden emotional consequences of low life satisfaction [53,54]. Older adults who evaluate their lives negatively are more likely to experience chronic worry, internalized tension, and perceived helplessness—affective states that directly undermine the physiological conditions required for restful sleep [29,44]. Rather than simply being a byproduct of poor life satisfaction, anxiety appears to be a primary conduit through which dissatisfaction may be linked to sleep disturbances [24].

This pathway is especially concerning because anxiety not only affects sleep latency and fragmentation, but also perpetuates a self-reinforcing cycle of nocturnal arousal and next-day fatigue, which may further lower mood and increase dissatisfaction [55]. The strength of this anxiety-based pathway, relative to the resilience pathway, suggests that the erosion of emotional security may outweigh the benefits conferred by psychological strengths alone. In other words, reducing emotional distress may be more urgent for improving sleep quality than enhancing positive psychological resources in certain vulnerable subgroups.

From an intervention standpoint, these findings highlight the need to integrate emotional regulation and anxiety-reduction strategies—such as cognitive behavioral therapy or mindfulness-based stress reduction—into sleep health programs for older adults [56]. Addressing anxiety as a modifiable mediator may produce greater marginal gains than resilience enhancement alone, particularly among individuals with co-occurring medical burdens or limited social resources.

An important contribution of this study lies in examining chronic disease as a contextual vulnerability factor that modifies the psychological pathways linking life satisfaction to sleep quality. The interaction patterns suggest that the association between anxiety symptoms and sleep quality is more negative among older adults with chronic disease. In other words, among older adults with chronic illnesses, the negative association between anxiety and sleep quality appears to be more pronounced [32]. This pattern may reflect the compounding of physical and emotional stressors; for example, persistent somatic discomfort, heightened illness-related vigilance, and the cognitive burden of managing complex treatments may render anxious arousal more difficult to downregulate at night [55,57].

Crucially, this moderating relationship did not extend to the resilience pathway. The strength of the association between psychological resilience and sleep quality remained relatively stable regardless of chronic disease status [10]. This divergence implies that while physical illness may exacerbate vulnerability to emotional dysregulation (i.e., anxiety), it does not necessarily diminish the buffering capacity of psychological strengths [58]. Older adults with high resilience may still mobilize adaptive coping even under conditions of health deterioration, maintaining a protective relationship with sleep quality [59]. These findings highlight the potential of targeting resilience as a stable psychological buffer, even among individuals experiencing somatic decline, and call for differential intervention strategies tailored to the interplay of mental and physical health conditions in aging populations [52].

Theoretically, this study advances a dual-process psychological model of sleep quality in later life by simultaneously incorporating protective (resilience) and risk-related (anxiety) pathways within a unified life satisfaction framework. By demonstrating that life satisfaction may be associated with sleep quality through divergent emotional mechanisms, the findings expand the boundaries of positive psychology theory in the geriatric context and reaffirm the value of integrative models that account for both resource enhancement and emotional vulnerability [54]. Moreover, the moderated mediation structure, anchored in health vulnerability theory, illustrates the importance of contextualizing psychological processes within broader physiological realities, particularly in older populations with high disease burden [60].

Practically, the study identifies several leverage points for sleep-focused interventions. First, enhancing life satisfaction should be recognized as a foundational strategy to promote sleep health. Interventions that foster positive self-appraisal, social connectedness, and goal engagement may yield downstream benefits for sleep regulation [61,62]. Second, the findings support the implementation of resilience-enhancing programs, including cognitive-behavioral training, mindfulness-based interventions, and social skills development, which may help older adults maintain psychological equilibrium and reduce susceptibility to stress-induced sleep disruption [56,57]. From a geriatric care perspective, routine screening of resilience-related coping capacity and anxiety symptoms may help identify older adults at higher risk of poor sleep, especially those with chronic disease [63]. Clinicians and community care providers may consider incorporating resilience-building strategies, such as coping skills training, problem-solving, and guided social engagement, into chronic disease management programs [64]. Targeted anxiety management, including brief cognitive-behavioral and relaxation-based approaches, may also be beneficial for chronically ill older adults [65]. Integrating these approaches with routine medical care may further improve sleep-related outcomes. Finally, given the heightened vulnerability of chronically ill individuals to anxiety-induced sleep problems, integrative interventions that combine medical management with emotional regulation training are urgently needed [32]. Tailoring these programs to disease-specific anxieties—such as fear of disease progression or treatment failure—may further enhance their effectiveness [57].

Collectively, this study underscores the need for personalized and psychologically informed approaches to sleep promotion in aging populations. By aligning intervention strategies with both psychological profiles and physical health status, public health programs may more effectively support healthy aging and reduce the long-term consequences of sleep disturbances among older adults [66,67].

This study has two main limitations. First, its cross-sectional design restricts causal inference, making it difficult to establish the temporal ordering among life satisfaction, psychological mediators, and sleep quality; future longitudinal research is needed to validate the proposed pathways and explore potential bidirectional effects. Second, all variables were self-reported, which may introduce response bias and shared method variance; moreover, sleep quality and life satisfaction were assessed using single-item measures, and psychological resilience was constructed from CLHLS items rather than a standardized resilience scale, which may limit measurement precision. In addition, pain associated with chronic illness may influence sleep quality, but a comprehensive measure of overall bodily pain was not available in the dataset; thus, residual confounding cannot be ruled out. Future studies incorporating objective sleep measures and clinically validated assessments of mental health would enhance the robustness and interpretability of the findings.

## 5. Conclusions

Drawing on a nationally representative sample of older adults in China, this study examined psychological pathways that may underlie the association between life satisfaction and sleep quality. Life satisfaction was associated with sleep quality, with psychological resilience and anxiety symptoms accounting for part of this association. Although chronic disease strengthened the association between anxiety symptoms and sleep quality, the index of moderated mediation was not significant. Therefore, these findings should be interpreted as associative rather than causal, especially given the cross-sectional design. These findings highlight the dual role of protective and risk-related psychological mechanisms in shaping sleep outcomes and underscore the importance of considering individual health status in psychosocial models of aging. Interventions aiming to improve sleep in later life should prioritize both emotional regulation and resilience-building strategies, particularly for those with chronic health conditions.

## Figures and Tables

**Table 1:** Descriptive statistics and correlation analysis.

	Sleep Quality	Psychological Resilience	Life Satisfaction	Anxiety	Chronic	M	SD
sleep quality	1					3.52	0.988
psychological resilience	−0.111 **	1				−0.374	0.681
life satisfaction	−0.258 **	0.131 **	1			2.09	0.81
anxiety	−0.281 **	0.047 **	0.186 **	1		−0.036	0.943
chronic	−0.009	−0.044 *	0.012	0.019	1	0.86	0.35

* *p* < 0.05, ** *p* < 0.01.

**Table 2:** Analysis of mediating relationship of psychological resilience.

Outcome	Predictor	R	R^2^	F	β	LLCI	ULCI	t
Panel A. Outcome: Psychological resilience		0.205	0.042	16.830 ***				
	Constant				−0.766 ***	−0.917	−0.615	−9.96
	Life satisfaction				0.101 ***	0.070	0.131	6.52
Panel B. Outcome: Anxiety		0.289	0.083	35.030 ***				
	Constant				0.216 *	0.012	0.421	2.07
	Life satisfaction				0.154 ***	0.113	0.195	7.34
Panel C. Outcome: Sleep quality (Total association)		0.301	0.091	38.390 ***				
	Constant				4.006 ***	3.792	4.218	36.85
	Life satisfaction				−0.286 ***	−0.328	−0.243	−13.09
Panel D. Outcome: Sleep quality (Direct association, with mediators)		0.375	0.141	50.320 ***				
	Constant				3.980 ***	3.769	4.190	37.02
	Life satisfaction				−0.240 ***	−0.282	−0.198	−11.14
	Psychological resilience				−0.099 ***	−0.148	−0.051	−4.01
	Anxiety				−0.232 ***	−0.268	−0.196	−12.69

Notes: Covariates were included in all models. * *p* < 0.05, *** *p* < 0.001.

**Table 3:** Mediated model tests with moderation.

Outcome	Predictor	R	R^2^	F	β	LLCI	ULCI	t
Panel A. Outcome: Psychological resilience		0.205	0.042	16.830 ***				
	Constant				−0.392 ***	−0.543	−0.241	−5.09
	Life satisfaction				0.101 ***	0.070	0.131	6.53
Panel B. Outcome: Anxiety		0.289	0.083	35.031 ***				
	Constant				0.252 *	0.047	0.456	2.41
	Life satisfaction				0.154 ***	0.113	0.195	7.34
Panel C. Outcome: Sleep quality		0.377	0.142	39.242 ***				
	Constant				4.028 ***	3.819	4.236	37.87
	Life satisfaction				−0.241 ***	−0.284	−0.199	−11.21
	Psychological resilience				−0.100 ***	−0.149	−0.052	−4.04
	Anxiety				−0.231 ***	−0.266	−0.195	−12.62
	Chronic disease				0.030	−0.065	0.125	0.62
	Int_1				0.047	−0.096	0.189	0.64
	Int_2				−0.121 *	−0.222	−0.021	−2.36

Notes: Int_1 = Psychological resilience × Chronic disease; Int_2 = Anxiety × Chronic disease. Covariates were included in all models. * *p* < 0.05, *** *p* < 0.001.

## Data Availability

The datasets analyzed during the current study are available in the National Archive of Computerized Data on Aging (NACDA) repository, persistent web link: https://www.icpsr.umich.edu/icpsrweb/NACDA/series/487 (accessed on 29 January 2026).
